# Partial trisomy 9p and partial monosomy 7p of an infant inherited from maternal balanced translocation: a case report

**DOI:** 10.1186/s12887-023-03986-3

**Published:** 2023-04-13

**Authors:** Rui Li, Chaojie Wang, Zhenhua Zhang, Dongxiao Li, Lifeng Li, Ding Zhao, Zhaojie Xu

**Affiliations:** 1 Henan Key Laboratory of Children’s Genetics and Metabolic Diseases, Children’s Hospital Affiliated to Zhengzhou University, Henan Children’s Hospital, Zhengzhou Children’s Hospital, Zhengzhou, 450018 China; 2 Department of Anal and Intestinal Surgery, Zhengzhou People’s Hospital, Zhengzhou, 450000 China

**Keywords:** Chromosome translocation, Copy number variation, Karyotyping, Partial monosomy 7p, Partial trisomy 9p

## Abstract

**Background:**

Subchromosomal deletions and duplications are the leading cause of congenital malformations and mental retardation in children. With the recent clinical application of genomic microarrays in the evaluation of patients with developmental delays and congenital malformations, it has led to the discovery of several new microdeletion and microduplication syndromes. However, there are no published reports involving patients with both microduplications in the 9p21.1-p24.3 region and microdeletions in the 7p22.1-p22.3 region.

**Case presentation:**

We report an infant with an autosomal abnormality confirmed by conventional karyotype combined with copy number variations sequencing (CNV-seq), showing the patient with an unbalanced translocation. The karyotype of the patient was 46, XX, der (7)t (7;9) (p22; p21) and CNV-seq results showed an approximately 32.34-Mb duplication in 9p21.1-p24.3 (200000-32540000) and an approximately 3.3-Mb deletion in 7p22.2-p22.3 (40000-3340000).

**Conclusions:**

The patient carried an unbalanced translocation 46, XX, der (7)t (7;9) (p22; p21) derived from her mother. The clinical presentation is closely related to the size and position of the missing and duplicated chromosomes. To our knowledge, the simultaneous occurrence of de novo partial trisomy 9p(9p21.1-p24.3) and partial monosomy 7p (7p22.2-p22.3) has not previously been reported up until now. The present study additionally demonstrated that CNV-seq combined with karyotype is able to reliably detect unbalanced submicroscopic chromosomal aberrations.

## Background

Trisomy 9p syndrome is a rare disorder, first reported by Rethoré et al. [1] in 1970. Most reported cases of trisomy 9p are accompanied by partial deletions of other chromosomes. It is characterized by multi-organ system involvement, including craniofacial anomalies, cardiac, genitourinary, skeletal and central nervous system (CNS) abnormalities [2]. Karyotype analysis is the “gold standard” for diagnosing chromosomal aberrations. It usually detects abnormal chromosome numbers and structural abnormalities such as deletions, duplications, translocations and inversions of large segments of 5–10 Mb or more, but not deletions and duplications of small chromosomal segments [3, 4]. With the development of molecular genetic techniques, the CNV-seq technique can detect micro-repeats and micro-deletions as small as tens of kilobases, and can determine the size of duplicated or missing fragments and their location on chromosomes, which is a powerful complement to the traditional karyotype analysis. In this study, we combined karyotype analysis of chromosome G and CNV-seq to perform cytogenetic and molecular genetic tests in a patient with growth retardation and mental retardation with congenital multiple malformations. in order to identify the origin of chromosomal abnormalities and analyze the relationship between chromosomal structural abnormalities and clinical phenotypes, thus providing a strong basis for clinical diagnosis and genetic counseling.

## Case presentation

The proband was a 4-month-old female born to a 29-year-old father and a 27-year-old mother via vaginal delivery at 38 gestational weeks. During pregnancy, no specific problems were identified. The patient was hospitalized in a local hospital for 7 days after birth for “respiratory distress syndrome”. She was found to have slow weight gain since birth, with a birth weight of 2.5 kg and a current weight of 3.5 kg, accompanied by poor feeding, dry vomiting, minor crying, and bruised lips while crying. In order to seek further medical treatment, the patient was admitted to Children’s Hospital Affiliated to Zhengzhou University (Zhengzhou, China) at 4-months-old for “malnutrition”. Since the onset of the disease, the patient had poor mental response, poor appetite, and slightly dilute stool. The examination showed a stunted development, malnutrition, and thin subcutaneous fat. There were peculiar facial features including wide eye spacing, small jaw, high palatal arch, left eyelid ptosis, hawkish nose and low ear position. The left thumb was attached on top of the palm, and the little fingers on both hands were flexed and deformed. The breath sounds of both lungs were coarse and a grade 3/6 murmur could be heard in the precordial region. Congenital heart disease was suspected. This diagnosis was followed by transthoracic atrial septal defect closure and arterial catheterization for treatment.

Peripheral blood samples were obtained from the patient, her sister and the parents for examination of chromosomes by metaphase G-banding and CNV-seq. The Children’s Hospital Affiliated to Zhengzhou University Ethics Committee approved the sample collection procedures and the family gave written informed consent. Chromosome karyotype analyses under sterile conditions was conducted on cultured lymphocytes according to standard protocols. Colchicine was added after 72 h of culture and cells were harvested after 1 h, after which they were filmed, G-banded for color development, photographed with a GSL120 fully automated scanner. 20 cells were counted and 5 karyotypes were analyzed by applying karyotype analysis software. Chromosomal karyotypes were determined according to the International System of Human Cytogenetics Nomenclature ISCN (2020). CNV-seq assays standard procedures were used isolate the genomic DNA of the proband and the parents from whole blood using PerkinElmer Chemagic 360 fully automated nucleic acid extractor. The library was constructed using the “Rapid PCR-free library construction technology” (Berrygenomics, Inc., Beijing, China). Detection of copy number variations (CNVs) was conducted by NextSeq CN500 (Illumina, Inc., USA) high-throughput sequencer. The sequencing type was single-end 36-base sequencing. The measured sequence fragments were compared to the known human reference genome (hg19). Analysis was performed using Konoan data analysis software (Berrygenomics Genetic Diagnostics, Inc., Hangzhou, China), and CNVs were detected with a resolution of 100 kb or more. The copy number variations were compared with the Database of Genomic Variants (DGV), the Database of Genomic Variantion and Phenotype in Humans using Ensembl Resources (DECIPHER), Online Mendelian Inheritance in Man (OMIM), and The Clinical Genome Resource (ClinGen) to annotate the reported disease-causing genes by comparison and analysis. According to the American College of Medical Genetics and Genomics (ACMG) guidelines and the CNVs diagnostic guidelines, CNVs are rated as 5 levels of risk: pathogenic, possibly pathogenic, benign, probably benign, and of unknown significance [5, 6].

**Fig. 1:**
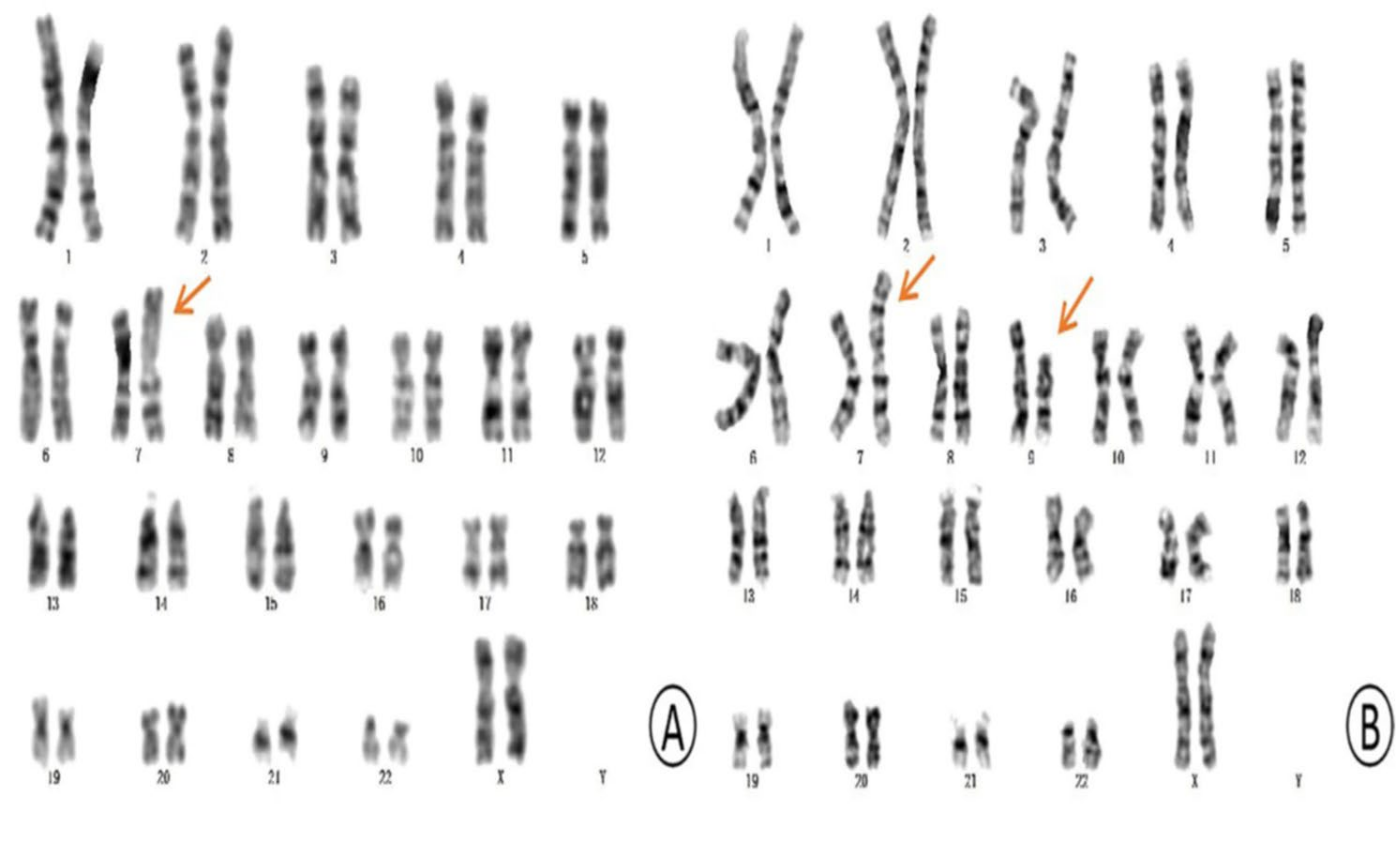
The results of karyotype analysis of chromosomes. (A)Karyotype of the patient. The karyotype of the patient indicated an abnormal karyotype: 46, XX, der (7)t(7;9) (p22; p21) mat. The arrow indicates the derived chromosome 7. (B)Karyotype of the mother of the patient. The karyotype of the mother of the patient indicated an abnormal karyotype: 46, XX, der (7)t(7;9) (p22; p21). The arrows indicated chromosomes with balanced translocation

**Fig. 2:**
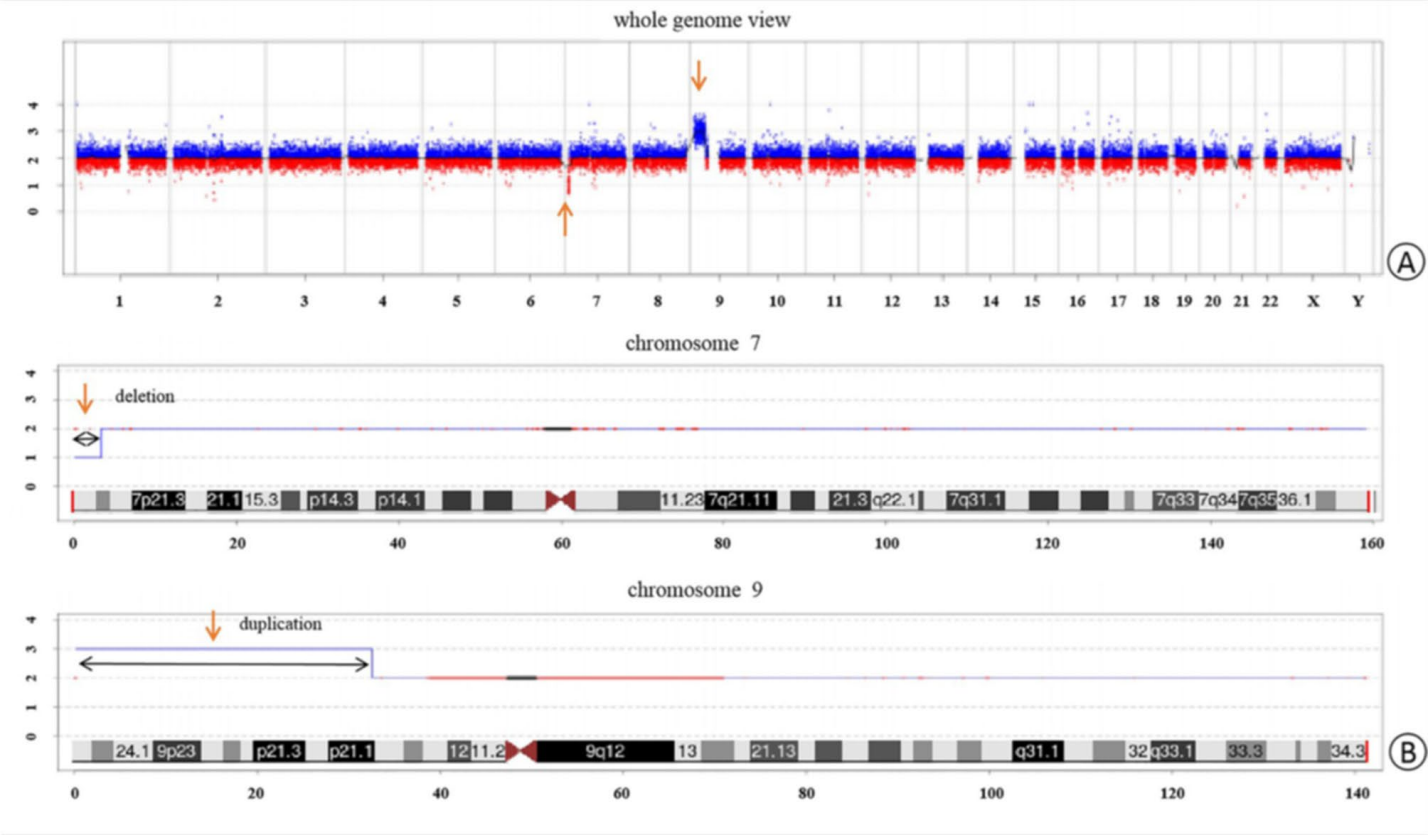
The results of CNV-seq analysis. (A)The whole genome view. (B)The CNV-seq results of the patient showed a 3.30-Mb deletion (40000-3340000) in 7p22.3-p22.2 and a 32.34-Mb duplication (200000-32540000) on the chromosome in 9p21.1-p24.3. The arrows indicate the breakpoints

The karyotype of the patient was 46, XX, der (7) t (7;9) (p22; p21) mat (Fig. 1A). The karyotype of the patient’s mother indicated a balanced translocation karyotype: 46, XX, t (7;9) (p22; p21) (Fig. 1B). Her sister and father exhibited a normal karyotype. The CNV-seq analysis revealed a 32.34 Mb duplication in the 9p21.1p24.3 (200000-32540000) (hg19) region, involving 100 OMIM genes, and a 3.30 Mb deletion in the 7p22.2p22.3 (40000-3340000) (hg19) region, involving 30 OMIM genes (Fig. 2A and B). By searching databases such as Decipher, OMIM, DGV, and ClinGen, clinical phenotype matching and interpretation of genetic patterns were performed for cases that had been reported in the databases. The results showed that the two CNVS of the child were reported in various databases and involved many genes. Finally, according to the ACMG guidelines and the guidelines for the diagnosis of CNVs, they were both classified as pathogenic CNVs.

## Discussion and conclusions

The present case report demonstrated that the patient carried an unbalanced translocation inherited from the mother who was a balanced translocation carrier, which resulted in partial trisomy for 9p (spanning ~ 32.34 Mb) and partial monosomy for 7p (spanning ~ 3.30 Mb). To the best of our knowledge, the present study is the first report of an unbalanced translocation involving chromosomes 7p and 9p.

9p trisomy is often caused by heterozygous segregation of familial chromosomal translocations. Most reports include deletions of other chromosomes. Common phenotypes of trisomy 9p include growth and language intellectual disability, abnormal ear position, hypertelorism, bulbous nose, low mouth angle, and abnormal hand and foot finger development[2]. The severity of the partial trisomy 9p phenotype was correlated with the length of the repeat in the short arm of chromosome 9 and the repeat region. Duplications in the 9p13-p21 region have less effect on mental development whereas some genes associated with mental development (*DOCK8*, *FOXD4*, *VLDLR*, etc.) are present in the 9p22-9p24 region where the very low density lipoprotein receptor gene (*VLDLR*) transduces a variety of extracellular signals across the neural cell membrane into the CNS, regulates synaptic plasticity and is important for specific learning and memory functions in the hippocampus [7]. The duplications in p21.1-p24.3 of chromosome 9 in the patient involved“trisomy 9 syndrome, which contains 100 OMIM genes. There are multiple patients in the Decipher database carrying pathogenic or potentially pathogenic variants that partially overlap with this CNV interval. It has been reported in the literature that the main clinical manifestations of patients with 9p22-p24 duplication include short stature, microcephaly, peculiar facial features, and congenital heart disease [8–10]. The clinical phenotype of this patient was generally consistent with the phenotypes reported in the literature, with the addition of other typical phenotypic features, such as a hawkish nose and presence of unilocular ptosis. This discrepancy may be due to the fact that it carries a 9p repeat fragment that is less consistent with the above literature. Another possibility may be due to its coexistence with a heterozygous deletion of approximately 3.30 Mb in the 7p22.2p22.3 region, which contains 30 OMIM genes including 11 morbidity-associated genes (*BRAT1*, *FAM20C, EIP3B*, *LFNG*, *INTS1*, etc.). *INTS1* and *BRAT1* genes are located at 7p22.3 and are associated with uniform neurodevelopmental disorders [11]. It has been reported in the literature [12] that the main clinical phenotype of patients with 7p22.2p22.3 deletion is a peculiar facial appearance, with developmental delay in speech, etc. Both chromosomal copy number variants in this patient have been reported frequently in patient databases, with the involvement of additional genes, and all were determined to be pathogenic CNVs according to ACMG guidelines. The reported phenotypes correlate with the patient’s phenotype, but the reported phenotypes were more variable and our patient was comparatively younger. Many phenotypes require further clinical excavation, verification and follow-up observations. Whether haploinsufficiency of any OMIM gene necessarily leads to a clinical phenotype requires further summary and follow-up of additional cases. In this case, the patient had both trisomy 9p and monosomy 7p. It is possible that abnormal alterations in these two chromosomes interact to form a specific phenotype.

Phenotypic outcomes such as recurrent spontaneous abortion, embryonic arrest and multiple neonatal malformations tend to manifest in carriers of chromosomal balanced translocation. The results of karyotype analysis suggested that the mother of the patient was a 46, XX, t (7; 9) (p22; p21) balanced translocation carrier, which was the direct cause of the microdeletion of segment 7p22.2p22.3 and the duplication of segment 9p21.1p24.3 in the patient. The reason for this is that the probability for a balanced translocation carrier to produce normal gametes is extremely low, with a theoretical probability of obtaining phenotypically normal offspring of only 1/9 [13, 14] and an actual probability of about 1/3. The mechanism of CNV formation in this patient may be due to the instability of the parental translocation chromosome break sites. For such children, prenatal diagnosis and preimplantation genetic diagnosis (PGD) are the main ways to reduce birth defects, so that children with genetic defects and various congenital anomalies can be detected early, and intrauterine treatment can be performed at the right time for those who are eligible and can be corrected, and those who cannot be corrected can have their pregnancies terminated in time to reduce the birth of defective children. In addition, prenatal diagnosis allows the chromosomes of both parents to be known in order to obtain the karyotype, breakpoints, and mode of inheritance of the translocation, providing a basis for genetic counseling and prenatal diagnosis for the incidence of translocation chromosome carriers and pregnancy outcome as well as revealing the genetic etiology of the clinical manifestations of the affected children. During the genetic counseling process, a comprehensive analysis should be performed in conjunction with several factors such as the type of translocation in the translocation carrier, the chromosomes involved in the translocation, and the location of the breakpoint of the translocation.

In conclusion, the occurrence of concurrent partial trisomy 9q (9p21.1p24.3) and partial monosomy 7p (7p22.2p22.3) has not previously been reported up to now. This study combined the application of karyotype analysis and CNV-seq to finally confirm the diagnosis for the patient. The use of CNV-seq and karyotype may facilitate a sensitive and powerful approach towards the diagnosis of submicroscopic unbalanced genomic rearrangements. This study clarified the origin and formation mechanism of CNV in children, and analyzed the relationship between chromosomal structure abnormalities and patient phenotype. Due to the clear mechanism of its occurrence and high risk of recurrence, clinical genetic counseling was presented to the patient’s mother where she was advised to undergo prenatal examination and diagnosis in the event of future pregnancies.

## Data Availability

The datasets that support the conclusions of this article are available by request to the corresponding author. We do not make participants’ data publicly available due to data protection restrictions and participant confidentiality.

## Abbreviations

CNV-seq: Copy number variants sequencing
CNS: central nervous system
DGV: Database of Genomic Variants
DECIPHER: Database of Genomic Variantion and Phenotype in Humans using Ensembl Resources
OMIM: Online Mendelian Inheritance in Man
ClinGen: Clinical Genome Resource
ACMG: American College of Medical Genetics and Genomics
